# Developments in PDT Sensitizers for Increased Selectivity and Singlet Oxygen Production

**DOI:** 10.3390/ma8074421

**Published:** 2015-07-20

**Authors:** Nahid Mehraban, Harold S. Freeman

**Affiliations:** Fiber & Polymer Science Program, North Carolina State University, Raleigh, NC 27695-8301, USA

**Keywords:** photodynamic therapy, tumor selectivity, photosensitizer, polymeric micelles, singlet oxygen production, amphiphilic, drug delivery, nanoparticles, triplet photosensitizer

## Abstract

Photodynamic therapy (PDT) is a minimally-invasive procedure that has been clinically approved for treating certain types of cancers. This procedure takes advantage of the cytotoxic activity of singlet oxygen (^1^O_2_) and other reactive oxygen species (ROS) produced by visible and NIR light irradiation of dye sensitizers following their accumulation in malignant cells. The main two concerns associated with certain clinically-used PDT sensitizers that have been influencing research in this arena are low selectivity toward malignant cells and low levels of ^1^O_2_ production in aqueous media. Solving the selectivity issue would compensate for photosensitizer concerns such as dark toxicity and aggregation in aqueous media. One main approach to enhancing dye selectivity involves taking advantage of key methods used in pharmaceutical drug delivery. This approach lies at the heart of the recent developments in PDT research and is a point of emphasis in the present review. Of particular interest has been the development of polymeric micelles as nanoparticles for delivering hydrophobic (lipophilic) and amphiphilic photosensitizers to the target cells. This review also covers methods employed to increase ^1^O_2_ production efficiency, including the design of two-photon absorbing sensitizers and triplet forming cyclometalated Ir(III) complexes.

## 1. Introduction

Photochemotherapy of cancer is often called photodynamic therapy (PDT) [1]. PDT is a minimally-invasive treatment modality that has been shown effective for cancers characterized by early-stage superficial tumors, including lung, esophageal, gastric, and cervical cancers.

PDT has been used for cancer treatment since the 1990s utilizing Photofrin^®^. Some of the drawbacks with this first generation, FDA approved, PDT agent (a photosensitizer) are poor light absorption properties in the NIR region [2], skin sensitization as a side effect of PDT treatments, and the presence of a complex mixture of uncertain structures [3]. Consequently, the development of new PDT agents devoid of these shortcomings while meeting the vital criteria for PDT cancer treatment remains important. The PDT agents developed after Photofrin are known as second-generation PDT sensitizers and were the basis for our previous review [4]. The present review provides an overview of approaches to photosensitizer (PS) design for enhanced ^1^O_2_ production and delivery for PDT as well as the fundamentals of this exciting technology.

### 1.1. Photosensitizers (PSs)

Dyes and pigments absorb and reflect certain wavelengths of light, which leads to the perception of color. Electronically, the difference between PS molecules and other dye types is the ability of PSs to transfer the energy of absorbed light to molecules in the vicinity or be utilized for photochemical reactions [5]. PSs are either porphyrin or non- porphyrin based. Examples of porphyrin-based PSs include phthalocyanines, chlorins, bacteriochlorin, and purpurins [4,6]. Protoporphyrin IX is a porphyrin based photosensitizers obtained from δ-aminolevulinic acid (ALA) through a biosynthesis mechanism [7].

**Figure 1:**
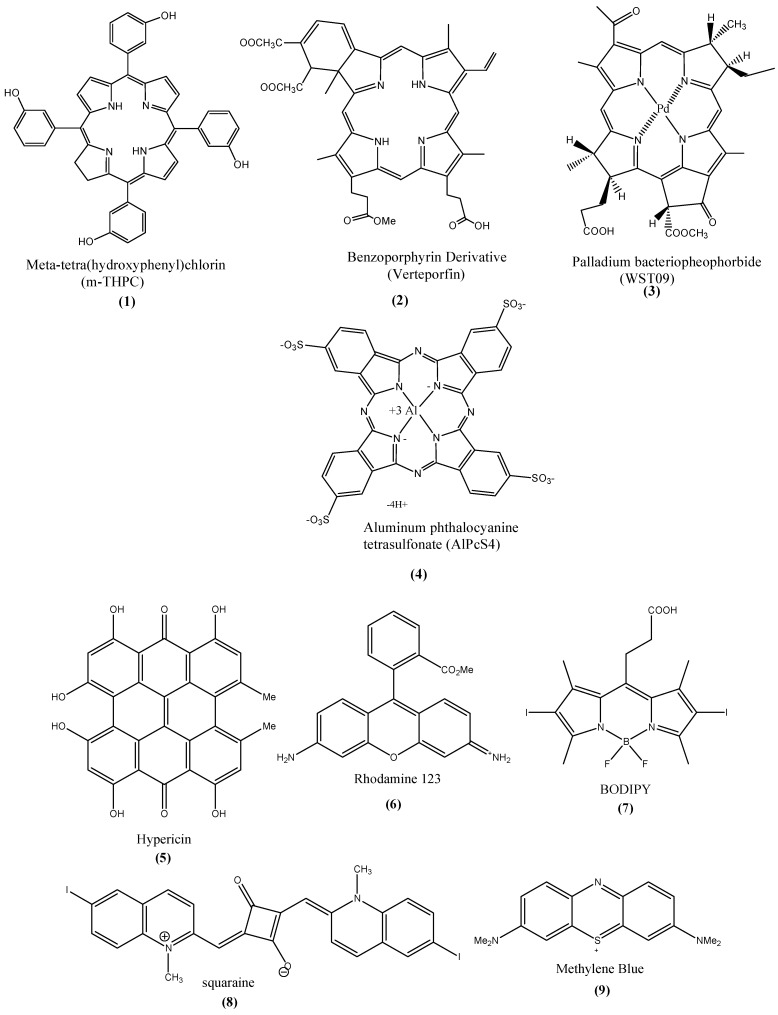
Examples of porphyrin (**1**–**4**) and non-porphyrin (**5**–**9**) PSs.

Non-porphyrin based PSs include psoralens, anthracyclines, hypericin. hypocrellins, cyanines, phenothiazinium compounds such as methylene blue, Nile blue analogs, toluidine blue, rhodamines, triarylmethanes, and acridines [2]. Two of the most recent developed PSs are bodipy [8] and squarines [9]. Examples of the porphyrin and non-porphyrin based PS types are shown in Figure 1.

Key target criteria for PDT PSs include:

1. High selectivity toward cancer cells
2. Production of toxic reactive oxygen species (e.g., ^1^O_2_) or free radicals
3. Optimal ADME (absorption, distribution, metabolism, excretion) [10]
4. Near IR light absorption
5. Absence of dark toxicity
6. High molar extinction coefficient

### 1.2. PDT Mechanism

**Figure 2:**
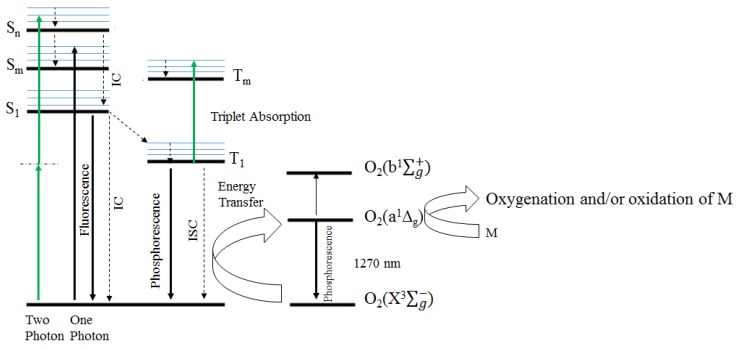
Formation of PDT reactive species through type I and type II photochemistry (modified from [12]).

PDT is based on the use of a PS, which efficiently populates an excited triplet state upon interaction with visible and NIR light. The triplet state of a PS (^3^PS*) can produce toxic reactive oxygen species (ROS), such as ^1^O_2_ or free radicals, by two different pathways. ^3^PS* can react with molecules to generate intermediate free radicals that in turn generate ROS (type I photochemistry). Alternatively, it can directly interact with molecular oxygen in its ground triplet state (^3^O_2_) to produce *in situ* cytotoxic ^1^O_2_ through an energy transfer process. This type II photochemistry (*cf.* Figure 2) is the most relevant PDT mechanism in cells, because most PSs are effective ^1^O_2_ producers [11]. The ROS generated are capable of causing irreversible damage if generated inside cells, particularly inside specific subcellular organelles (e.g., mitochondria) where PSs can localize and accumulate [11]. PDT would therefore be able to selectively kill diseased cells, provided that selective delivery of the PS to the target could be assured.

### 1.3. Singlet Oxygen Formation

The term singlet oxygen (^1^O_2_) refers to the two lowest energy excited electronic states of oxygen, O_2_(a^1^∆_g_ and O_2_(b^1^∑_g_^+^), as shown in Figure 2. O_2_(b^1^∑_g_^+^) is unstable in aqueous media, decaying rapidly to a^1^∆_g_ [13]. One way to form ^1^O_2_ involves energy transfer from an excited PS to ground state oxygen O_2_(X^3^∑_g_^−^). Energy transfer can also result in the production of O_2_(b^1^∑_g_^+^) which quickly decays to O_2_(a^1^∆_g_) in solution. ^1^O_2_ can form upon oxygen quenching of the S_1_ or T_1_ state of the PS but the most efficient ^1^O_2_ precursor is T_1_, which has a longer lifetime than S_1_ [12]. In general, the quantum yield of photosensitized singlet oxygen can be stated as:

ɸ_∆_ = S_1_ quenching + T_1_ quenching (1)

where S_1_ quenching reflects the amount of ^1^O_2_ produced by ground state oxygen quenching of the PS S_1_ state and T_1_ quenching reflects the amount of ^1^O_2_ produced by quenching of the PS T_1_ state [14].

A detailed and excellent discussion of the lifetime of intracellular ^1^O_2_ is given in the review of Ogilby [12]. A critical point is that data from single cell and ensemble studies indicate that ^1^O_2_ lifetime in a cell is significantly longer than long believed. Early on, time-resolved or photobleaching experiments were used to estimate intracellular ^1^O_2_ levels and gave values of 10–300 ns [11,15,16,17]. Since then, the development of methods for monitoring ^1^O_2_ from laser-induced phosphorescence, in time-resolved solution-phase experiments, led to lifetimes in H_2_O and D_2_O-incubated cells of ~3.5 µs and ~15–20 µs, respectively [17,18,19]. Clearly these values are much longer than long presumed.

Diffusion of ^1^O_2_ through surrounding medium can take place once ^1^O_2_ forms at sites where PS is localized. The distance, d, that ^1^O_2_ subsequently travels has been estimated from the following equation:

(2) $$d=\sqrt{6tD}$$

Thus, d depends on ^1^O_2_ lifetime τ_Δ_, the magnitude of diffusion coefficient D and time period t. Bearing in mind that ^1^O_2_ lifetimes of 2–3.5 µs in H_2_O and H_2_O incubated cells, t = 5τ_∆_ (10 μs) and a value of 4 × 10^−6^ cm^2^ s^−1^ for D were used to estimate the radial diffusion distance [12]. This led to d = 155 nm. At τ_∆_ ~25 μs, d = 550 nm was obtained. These results indicate that the long accepted diffusion limit of 10–20 nm from the site of ^1^O_2_ formation is no longer appropriate.

### 1.4. Mechanisms of Cell Death

While dependent on many factors, with the subcellular location of the PS being only one, PDT treatment can cause cell death by pathways involving apoptosis and necrosis. Necrosis is an unprogrammed cell death that is caused by chemical and physical damage [20]. It is a quick method of degradation that can affect widespread cell populations and can be characterized by cytoplasm swelling, damage to organelles, and disruption of the cell membrane. As a result of necrosis, intracellular contents will be released which leads to inflammation *in vivo*. Necrosis is more likely to happen when high doses of light are being used. Apoptosis is a programmed cell death that can be identified in single cells that are surrounded by other normal-looking cells and is characterized by cell shrinkage. Apoptotic cells tend to fragment into multiple membrane enclosed spherical vesicles, *in vitro*. These vesicles can be scavenged by phagocytes *in vivo*, and inflammation can be prevented in case of apoptosis and cells die in an immunologically-controlled way. [20]. Recent studies have shown that autophagy plays a role in PDT [21]. Autophagy is a process involving the transportation of cellular organelles and proteins through lysosomal degradation pathways and is related to cell survival, development, and differentiation. When proteins undergo irreversible ROS damage forming toxic oxidized protein, autophagy will be stimulated to remove these toxic species. Failure in these mechanisms leads to accumulation of oxidized macromolecules beyond the capacity of the cell to be repaired. In this case, the vital functions of the cells will be compromised which leads to cell death [22].

PSs differ in terms of pharmacokinetics and biodistribution. After their injection into the blood stream, they can bind to endothelial cells, the adventitia of the vessels, then accumulate within the tumor cells or bind to the extracellular matrix. The cytotoxicity induced by the absorbed light is restricted to the area of irradiation [23]. The effectiveness of PDT depends on the stage at which light has been delivered [24]. PDT can also lead to tumor death by deprivation of oxygen and nutrition arising from anti-vascular effects such as hemorrhage or thrombosis in tumor blood vessels [24].

### 1.5. Therapeutic Irradiation

**Figure 3:**
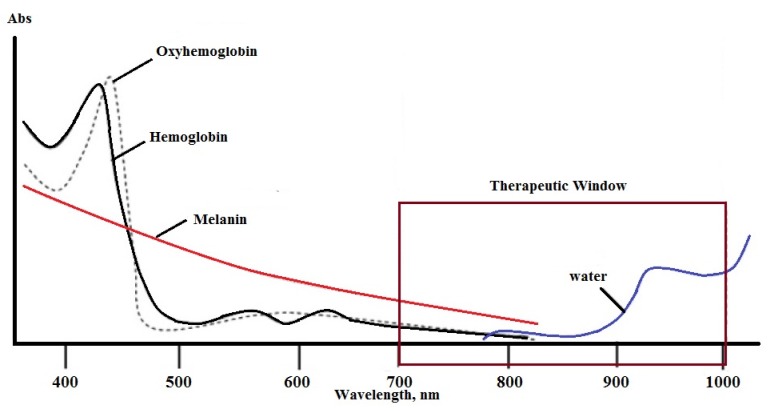
Illustration of the therapeutic window for PDT treatment.

To avoid undesired light absorption by biological chromogens instead of the PSs, the λ_max_ of the target PS should be >650 nm. Excitation at wavelengths in the near IR region also leads to deeper light penetration. This range of wavelengths is called the therapeutic irradiation window and is illustrated in Figure 3 [25]. Bearing in mind that the energy of the light decreases as wavelength increases, the preferred λ_max_ has been reported to be 630–800 nm by some [16] and 650–950 nm by others [26]. The molar absorptivity (ε_max_) of a PS should be >20,000 to minimize the required dose of the PS for PDT treatment [27].

### 1.6. Importance of Subcellular Localization

Controlling the subcellular localization of PS in cells is important because this has an impact on both PDT efficiency and the potential for cytotoxic affects. In this regard, it has been reported that even low levels of singlet oxygen generated in or near the mitochondria is far more efficacious than a large amount generated in the cell nucleus [28]. This opens the door to a pro-drug approach, *i.e.*, forming the PDT-active species at the target site, as an ideal way to minimized unwanted cytotoxic activity. An example is the formation of PpIX from ALA (5-aminolevulinic acid) in the mitochondria. It should be noted, however, that localization of PS in a subcellular structure does not guarantee that it will remain there. Depending on PS solubility and local concentration gradients it can diffuse out to the cytoplasm. Consequently, the design of PSs capable of bonding to the target site has been suggested [22].

It is also reported that low-level lysosomal photodamage can increase PDT efficiency due to subsequent mitochondrial photodamage. This phenomenon is not related to the amount of ROS formation, but appears to involve an increase in apoptotic signals arising from mitochondrial photodamage [29]. Localization of PS in mitochondria can be observed by using fluorescence imaging to monitor the coincidence of the PS and Rhodamine-123, taking advantage of the utility of Rhodamine-123 as a mitochondria specific stain.Examples of PSs targeting lysosome and mitochondria are listed in Table 1.

**Table 1:** Examples of PSs targeting lysosome and mitochondria.

Photosensitizer Type	Sub-Cellular Localization	Reference
*N*-aspartyl chlorin E6 (NPe6)	Lysosome	[29]
(Benzoporphyrin derivative) (BPD)	Mitochondria	[29]
5-Ethylamino-9-diethylaminobenzo [a]phenothiazinium chloride (EtBNS)	Lysosome	[29]
Galactose conjugate of 3-(1-hexyloxyethyl)-3- devinyl pyropeophorbide-a (HPPHgal)	Lysosome	[29]
Porphyrin-rhodamine B cation	Mitochondria	[30]
Porphyrin-mono-triphenyl phoosphonium cation	Mitochondria	[30]
Triphenylphosphonium (TPP) cation	Mitochondria	[31]
(E)-*N*-alkyl-4-[2-(ferrocenyl) vinyl]pyridinium cations	Mitochondria	[31]

**Figure 4:**
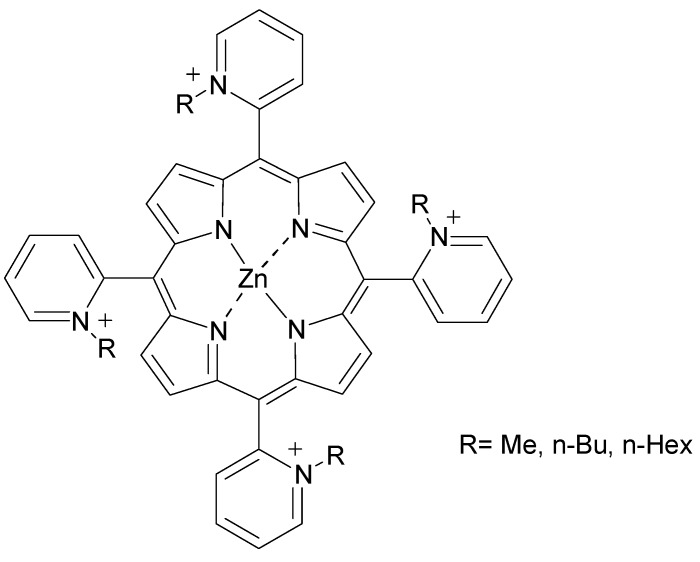
Structure of Zn(II) *N*-alkylpyridylporphyrins designed for mitochondria targeting.

Relatively new PS compounds targeting mitochondria include Zn(II) *N*-alkylpyridylporphyrins Figure 4. The presence of *n*-hexyl groups in these cationic compounds increased mitochondrial uptake and distribution. Localization in the vicinity of cytochrome c oxidase caused its inactivation during illumination. Light-induced inactivation accounts for the resultant mitochondrial photodamage and suppressed respiration and cell death [32].

## 2. PS Uptake and Selectivity

While the formation of ^1^O_2_ in the right subcellular location is essential to optimizing PDT efficiency, the proficient delivery of PSs to the right location is the essential starting point. Key considerations associated with achieving this goal are covered in this section.

### 2.1. Effect of Hydrophobicity (Lipophilicity) and Hydrophilicty on Cell Uptake and Selectivity

Arguably, one of the most critical issues in PDT sensitizer design is selectivity. The higher the selectivity, the higher the accumulation of the PS in cancer cells and the lesser the side effects for patients. PS selectivity is influenced by its hydrophobicity/hydrophilicity and can be expressed as the logarithm of the octanol/water partition coefficient (logP) as well as the degree of asymmetry that is present in the PS. Typically, a molecule with average size of the porphyrin, 2,3-dihydroxy-5,10,15,20-tetrakis(3-hydroxyphenyl) porphyrin, with logP of 9.3 can enter the cells upon diffusion through the phospholipid bilayer [33].

Most of the relatively hydrophobic PSs show sufficient affinity to bind to membranes. Even water soluble PSs such as *N*-aspartyl Chlorin e6(NPe6) and sulfonated porphyrins/phthalocyanines bind to the membrane because they also contain a large hydrophobic ring [34]. An example of an amphiphilic PS that accumulates in lysosomes and endosome *in vitro* and *in vivo* is AlPcS2a [15] and an example of a lysosomal PS is *N*-aspartyl chlorin e6 (NPe6). It has been demonstrated that PDT using lysosomal NPe6 on living human cell lung adenocarcinoma cell (ASTC-a-1), initiated a mitochondrial apoptotic pathway [35]. Temopofin, known by brand name of Foscan^®^, is a lipophilic sensitizer with logP of 5.5 that tends to aggregate and interact with plasma proteins [10] and cell membranes [36].

Hydrophilic as well as aggregated PSs will be taken up through pinocytosis and/or endocytosis and they will accumulate mostly in the lysosome and endosome. As a consequence of light exposures, lysosomes become permeabilized and the sensitizers and enzymes can be released into the cytosol. As a result, tubulin forms which leads to accumulation of cells in mitosis and causes cell death in some cases [37]. Accumulation of cationic PSs takes place mostly in mitochondria because of the transmembrane potential of the inner mitochondrial membrane [38]. Cationic PSs with a delocalized positive charge tend to localize in mitochondria with a high negative membrane potential of 150–170 mV [31].

One advantage of hydrophilic PSs is their low tendency to aggregate in aqueous media. Aggregation facilitates internal conversion to the ground state which suppresses ISC [39]. Another advantage of hydrophilic PSs is that they decompose or disappear from the body faster, which results in fewer side effects. Consequently, water solubility can increase bioavailability and *in vivo* distribution [40]. The disadvantage of hydrophilic PSs is the low penetration of these compounds through cell membranes compared to hydrophobic PSs. AlPcS is an example of a hydrophilic PS having low cell internalization efficiency [41]. Water soluble phenothiazinium compounds such as methylene blue not only have poor cell/tissue penetration, they also have low stability under reductive biological conditions [42].

Although hydrophobic (lipophilic) PSs have a higher tendency to permeate cell membranes, their tendency to undergo aggregation in aqueous media makes them non-ideal without using a suitable delivery system. Hydrophobic PSs also tend to remain longer in the patient’s body compared to hydrophilic PSs. These observations have caused PSs with amphiphilic properties through conjugation with water soluble or amphiphilic polymers or colloidal administrations to become attractive in PDT studies [43,44,45,46,47].

### 2.2. Effect of Intracellular pH on PS Uptake

**Figure 5:**
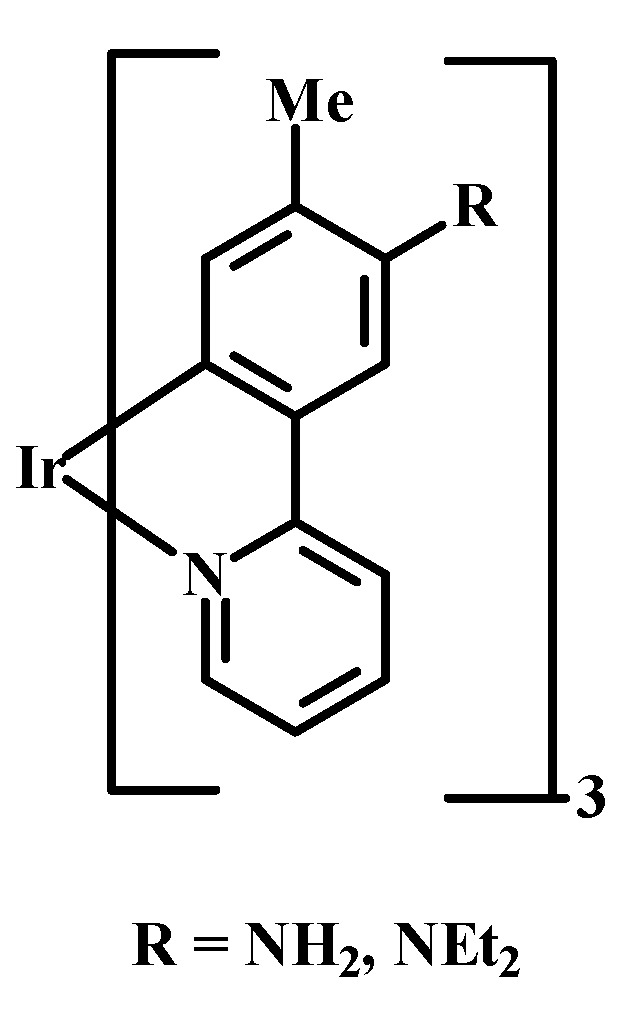
Examples of pH responsive Ir(III) complexes.

PS uptake into various organelles is also affected by internal pH. It is known that the pH of organelles such as the Golgi apparatus, endosomes, and lysosomes are 6.7, 6.5, and 5.5, values that are lower than the pH of the cytosol and mitochondria (7.2–7.5) in normal cells. In addition, it has been reported that the pH of some types of cancer tissues are in the slightly acidic range, viz. < 7.3. Interestingly, there are few examples of reversible on/off pH-probes that respond to changes in intracellular pH. Regarding pH probes that are sensitive to a change in intracellular pH, Ir-tolylpyridine complexes have been used to selectively stain lysosomes. For instance, a new pH-sensitive Ir(III) complex was developed that contains three *N*,*N*-diethylamino groups (Figure 5). The luminescence emission of the complex produced a band at 554 nm in DMSO, which was ~60 nm shorter than the analog having *R* = NH_2_. Protonation of the three NEt_2_ groups induced a considerable enhancement in the band at 497 nm. In aqueous media, the emission intensity of this complex at 494 nm was very weak at pH > 7.4 but was considerably enhanced at pH < 7. In a subsequent study, new pH-responsive Ir(III) complexes were developed, leading to ^1^O_2_ generation upon irradiation. The induction of necrosis-like cell death of HeLa-S3 cells upon irradiation of at 465 nm was reported [48,49].

### 2.3. Use of Nanoparticles

One approach to increasing PS selectivity involves using a molecule having high affinity toward cancer cells, such as folic acid [50,51] or antibodies [52], peptides, LDLs, and polymers [3]. This approach is employed for third-generation PSs wherein the affinity for cancer cells increases by targeting subcellular compartments such as mitochondria [53]. Therefore, third-generation PSs are often second-generation PSs bound to carriers for selective accumulation in tumors [54].

**Figure 6:**
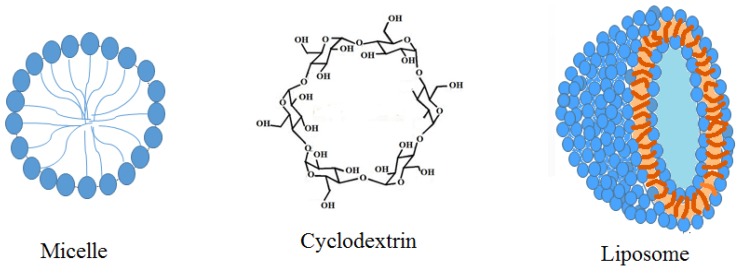

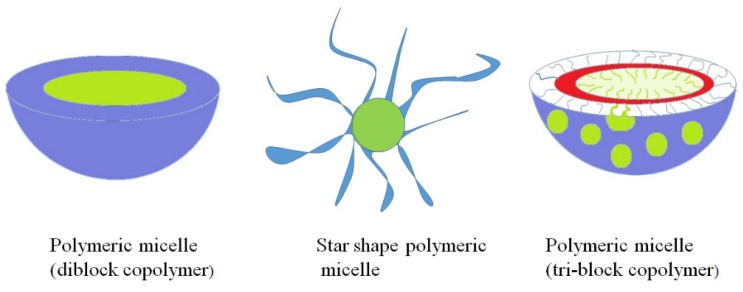
Examples of nanoparticle structures developed for PDT treatments.

The second approach involves using nanoparticle-based drug delivery methods in PDT. Although amphiphilic PSs or third-generation PSs can enhance selectivity, employing nanoparticles in PDT studies has become very attractive for the following reasons: (1) lower levels of the PSs can be used for PDT treatment; (2) PSs can be used in monomeric form within some of the nanoparticles; (3) most of the FDA approved PDT agents are hydrophobic and nanoparticles can increase the selectivity of these compounds to reduce side effects after treatment as well as aggregation problems; (4) dark toxicity would be less of a problem [55]; (5) exploiting strategies such as pH sensitivity, thermal sensitivity, peptide or antibody tags in nanoparticle system can increase selectivity more efficiently; (6) having control on making nanoparticles with a diameter of less than 200 nm for better passive targeting through EPR system; (7) increase in cell uptake [38,46]. Examples of some of the nanoparticles that have been used in PDT studies so far include liposomes [56,57], micelles, polymeric micelles, carbon nanodots [58], quantum dots, catanionic vesicles [59], gold nanoparticles [60,61], hydrogels [62], and dendrimer nano particles [63], TiO_2_[64], examples of which are shown in Figure 6.

#### Polymeric Micelle/Micelles

**Figure 7:**
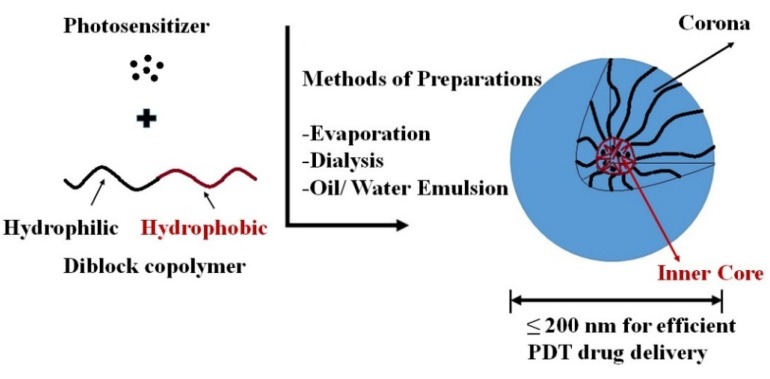
Schematic representation for spherical polymeric micelle formation via different methods. Co-solvent evaporation [68], dialysis [69], oil/water emulsion.

Polymeric micelles are made of amphiphilic block or graft copolymers that form spontaneously in aqueous media due to thermodynamically-favored aggregation above the critical micelle concentration (CMC) [65]. Micelles with lower CMC levels have higher thermodynamic stability, which can be obtained by increasing the hydrophobic section of the amphiphiles [65,66]. Below the CMC level polymer chains tend to remain in the monomolecular state. As their concentration in water increases, however, the hydrophobic component tries to escape water and forms the inner core. Subsequently, the hydrophilic head interacts with water on the surface of the spherical micelle and forms the corona. Formation of the micelle core is due mainly to hydrophobic interactions as well as hydrogen bonding of constituted block copolymer, metal complexation and electrostatic interactions [67]. A feature of polymeric micelle is illustrated in Figure 7.

**Figure 8:**
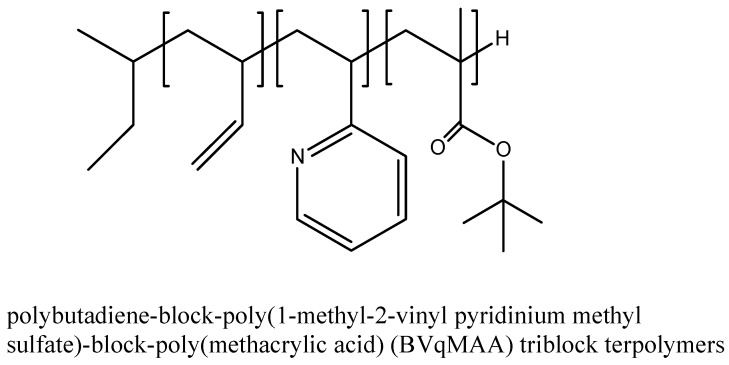
Examples of tri-block copolymers used to form polymeric micelles.

Having the hydrophobic core enables micelles to entrap hydrophobic PSs via secondary valency forces or electrostatic interactions. The use of nanoparticles such as polymeric micelles provides the framework for next generation PSs [70]. In addition to di-block copolymers, tri-block copolymers, Figure 8 [71], star-shaped polymers [68,72] can be used to form micelles. In some cases the polymer component is a polypeptide [73].

Studies in this area have taken into consideration that the PS employed may concentrate in the tumor cells via active and passive-targeting strategies [74]. Using artificial carriers such as cationic liposomes, silica nanoparticles, and polymeric conjugates can prolong the plasma circulation half-lives of the PS and increase its accumulation in tumors through enhanced permeability and retention effects [74,75].

The passive targeting mechanism takes advantage of an “enhanced permeation and retention” (EPR) process. EPR allows preferential extravasation of drugs though leaky vasculatures, due to having an incomplete endothelial barrier and poor lymphatic drainage in most of the tumor structure [76,77]. Stimuli-responsive polymeric micelles giving controlled release upon pH and temperature changes can be used to increase PS delivery to the specific target [78]. Salt concentration, magnetic fields, light [71], chemical auxiliaries, and enzymes [79,80] are types of stimuli than can be used. In addition to the EPR mechanism, active targeting facilitates penetration of the PS carrier into the cancer cells via surface functionalization of the nanocarrier with a specific molecular motif such as an antibody or peptide. EGFR-targeting peptide ligand GE11 has been used to target head and neck squamous cell carcinoma H&N SCC cells *in vivo* and *in vitro* [76]. Active targeting prevents one of the problems associated with passive targeting, which is nanoparticles accumulated in the tumor stroma passively can be redistributed into the blood stream, which reduces selectivity [81].

One of the advantages of polymeric micelles over standard micelles made from compounds such as polysorbate 80 (Tween 80^®^) is their higher stability in solution. Also, polymeric micelles can solubilize a larger amount of the PDT agent and they are suitable for parenteral administration [65]. Micelles can be made of amphiphilic peptides. These amphiphiles can form spherical to nanofiber structures due to hydrogen bonding and hydrophilic interactions. Using lysine as a sub-unit, the hydrophilic component will be ionized at acidic pH levels and the micelles become destabilized due to electrostatic repulsion interactions [80].

Other advantages of polymeric micelles include ease of preparation, efficient drug loading, controlled drug release, and extended blood circulation that allows efficient targeting through the EPR effect. This also reduces the undesirable bio-distribution of hydrophobic compounds [82]. Polymeric micelles are one of the nanocarriers that can resolve the hydrophobicity and/or toxicity drawbacks of many PSs [73]. Polymeric micelles with diameter size of 10–200 nm can be used for drug delivery because they are large enough to escape rapid renal clearance and small enough to avoid removal by reticuloendothelial clearance (RES). Therefore, permeability and retention (EPR) in tumor tissue will take place more effectively in vascular architecture that is leaky or defective around tumor cells [70,78]. Usually the hydrophilic end of the molecule is PEG and this forms small size micelles (10–100 nm) [83].

**Figure 9:**
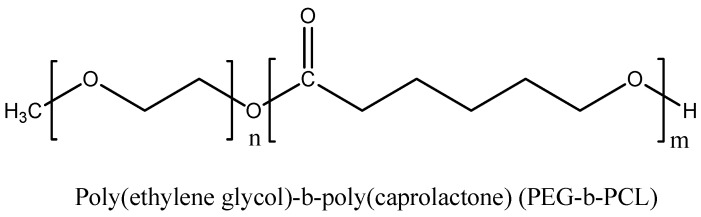
Structure of PEG-b-PCL.

One drawback of having long circulating polymeric micelles is unwanted photoactivity in normal cells. Li and co-workers showed that PEG-b-PCL, Figure 9, containing a generator and scavenger of ^1^O_2_ combination can reduce phototoxicity in normal cells [82].

**Figure 10:**
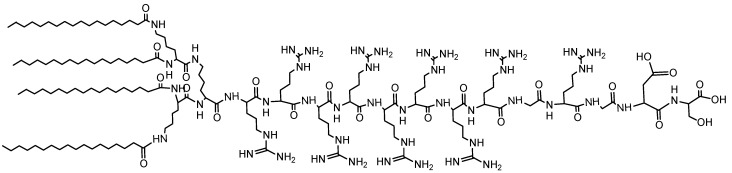
Structure of a surfactant-like tetra-tail amphiphilic peptide.

To increase the inherent biocompatibility and bioactivity of micelles, peptides can also be used. The problem with these types of micelles is a low hydrophobic tail interaction that decreases the stability of the micelles. This problem can be resolved by increasing the number of hydrophobic tails. Formation of a stable micelle is achieved by utilizing a surfactant-like amphiphilic peptide, Figure 10, with four hydrophobic tails [84].

**Figure 11:**
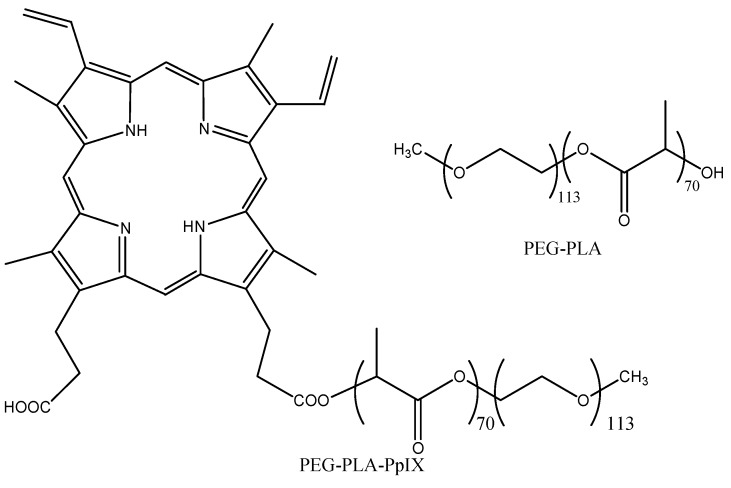
Conjugation of protoporphyrin IX to PEG-PLA.

Another advantage to using polymeric micelles is the ability to protect unstable PSs such as 5-ALA in aqueous media at neutral to basic pH. 5-ALA is a pro-drug that can be converted into protoporphyrin IX (PpIX) in proliferating cancer cells *versus* normal cells. 5-ALA is also very polar, which limits its diffusion through membranes. Conjugation of PpIX to PEG-PLA, Figure 11, increases PDT efficiency [85]. Hydrophobic PS conjugated to hydrophilic polymers can self-assemble into nanoparticles [86].

Controllable release is another advantage of using polymeric micelles. Trigger-controllable release of the PDT sensitizer by l-phenylalanine was reported for biodegradable supramolecular polymer micelles (SMPMs). In response to an additional l-phenylalanine molecule, SMPMs disassemble due to interactions between l-phenylalanine and sensitizer-loaded SMPMs [87]. To increase the stability of the polymeric micelles nanoparticle, it can be mineralized using calcium phosphate [73].

**Figure 12:**
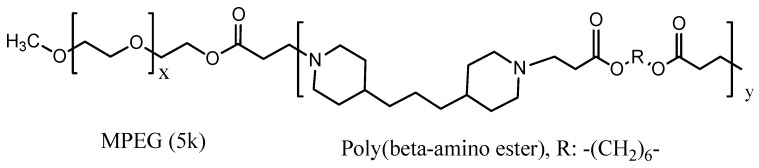
A pH sensitive MPEG poly(β-amino ester) structure for micelle formation.

One way to influence sensitizer release from polymeric micelles is to take advantage of the pH difference between the extracellular of normal cells *vs.* tumor tissue. The extracellular pH of tumor tissue is typically 6.4–6.8, due to accumulation of lactic acid [88], while the extracellular pH is 7.4 for normal cells. However, only a few pH-sensitive polymers are responsive to the extracellular pH of tumor tissue and others are sensitive to endosomal or lysosomal pH. pH-Responsive MPEG poly(β-amino ester) polymeric micelles are responsive to extracellular tumors. This type polymer micelle is pH responsive due to its tertiary amines [78], an example of which is shown in Figure 12.

**Figure 13:**
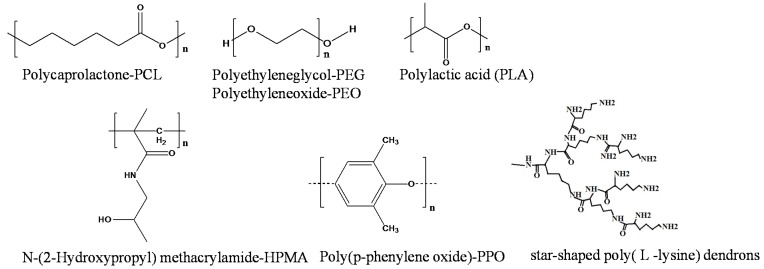
Examples of structural units reported in Table 2.

Some of the recent studies involving the use of polymeric micelles are summarized in Table 2, and examples of polymer structures are shown in Figure 13.

**Figure 14:**
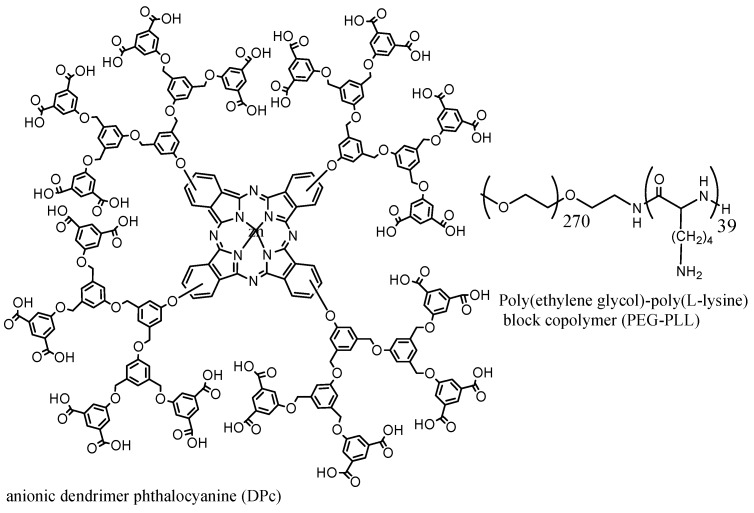
A dendrimer-based phthalocyanine structure for sensitizer delivery.

Utilizing DSPE-mPEG_2000_ micelles, 89% of hypericin was encapsulated and sub-localization into mitochondria occurred. Phototoxicity increased 2.5-fold compared to the case involving PEGylated PSs [89]. Drug loading efficiency for MPEG-poly(β-amino ester) block copolymer of 70%–80% was reported for PpIX [78]. Using PEO_750_-b-PCL5 polymer increased the cell uptake by 60% for phenophobide [70]. Loading capacity of encapsulated PpIX will increase from 0.2% to 4% by conjugation of the PpIX with PEG-PLA [85]. Highest loading capacity was observed using micelles containing benzoyl and naphthoyl end groups. The loading capacity for these micelles was 30% w/w [83]. Phototoxicity of dendrimer phthalocyanine encapsulated in PEG-PLL increased by 100-fold compared to the dendrimer phthalocyanine itself, Figure 14 [90].

Many studies using nanomedicine technologies have been conducted. However, translating these studies into clinical application will not be possible if the parameters needed to maximize cell killing were not reported. As an example, optimized or maximum cell internalization is reported as ≤ 2 h for A431 cells and takes place when a 10 mol% EGFR-PEG-PCL-GE11 component is used in micelle formulation [91].

**Table 2:** Examples of polymeric micelles/micelles tested *in vitro* and *in vivo* for PDT.

Strategy for Targeting & Drug Release	Sensitizer	Polymer/Monomer	Loaded Particle Size (nm)	*In vitro*/*In vivo*	Ref.
**Active** targeting	Silicon phthalocyanine	PEG-PCL-GE11 Peptide (YHWYGYTPQNVI)		Human epidermoid carcinoma and head & neck cancer	[76,81]
**Active** targeting Folate amino acid and overexpressed folate receptor	THPC	Poly (2-ethyl-2-oxazoline)-*b*-poly( d, l-lactide) (PEOz-PLA) (m-THPC-PM) (folate-m-THPC-PM)	103.8	*In vitro* on epidermoid cell line (KB); *In vivo* on KB xenograft mice	[78,92]
**Active** Galactosyl moiety for increased selectivity toward ASGP receptors on HepG2 cells	Porphyrin (conjugated to polymer)	APP-PAEMA_PCL Gal-APP-PAEMA-PCL	60	Human laryngeal carcinoma (HEp2); Human hepatocellular liver carcinoma (HepG2)	[69]
**Passive** Cellular endocytosis	Doxorubicin (DOX)	Star shaped poly(L-lysine) dendrons porphyrin polymer (PP-PLLD)	150–192.5	Human nasopharyngeal carcinoma (CNE2)	[72]
**Passive** targeting; Dual chemo-photodynamic therapy	Paclitaxel Synthetic chlorin	Star-shaped di-block copolymer (CSBC-58)	103.2	Breast cancer (MCF7)	[68]
**Passive** targeting	Pheophorbide a (phA) & β-carotene(CAR)	Poly(ethylene glycol)-b-poly(caprolactone) PEG-b-PCL	100	Human breast and cervical cancer cell line	[82]
**Passive** targeting	Chlorin e6	Poly(ethylene glycol)- b-poly(l-aspartic acid)-b-poly(l-phenylalanine) (PEG-PAsp-PPhe)	74.6	*In vitro* assay on MCF7	[73]
**Passive** targeting (endocytosis); Dual chemo-PDT	*m*-THPC SN-38	Chlorinated core star shape block copolymer (CSBC)	115.7–163.7	Human colon cancer (HT-29); *In vivo* xenograft	[77]
**Passive** targeting Dual photothermal (PTT) and PDT	IR825 Chlorin e6	C18PMH-PEG_-_Ce6-Gd	100–200	*In vitro* and *in vivo* assay on 4T1 Cell line	[93]
**Passive** endocytosis	Dendrimer phthalocyanine DPc	DPc + (PEG-PLL) → (DPc/m)	50	A549 cells in mice	[90]
**Passive** targeting Accumulate in mitochondria	Hypericin	DSPE-mPEG_2000_ micelle	12	Malignant brain tumor (MBTs)	[89]
Enzyme control release (in presence of l-phenylalanine)	THPP	Ethyl cellulose-graft-poly(ε-Caprolactone) and alpha cyclodextrin → EC-g-PCL and α-CD	205	85% THPP release in 6 h; MCF-7	[87]
**Passive** pH responsive polymeric micelles	PpIX	MPEG-Poly(β-amino ester) block copolymer (PpIx-pH-PMs)	122	*In vivo* on live SCC7 tumor-bearing mice; *In vitro* on SCC7 cells	[78]
**Passive** targeting	Pheophorbide	PEO_750_-b-PCL5 polymer	20	Human breast cell line (MCF-7)	[70]
**Passive** targeting	Porphyrazine	Polybutadiene-block-poly(1-ethyl-2-vinyl pyridinium methyl sulfate)-block-poly(methacrylic acid) (BVqMAA) triblock terpolymers	256	*In vitro* assay on A549 cells; Diameter size 6%–12% loading; *In vivo* assay	[71]
**Passive** targeting	Chlorin e6/Fe_3_O_4_	Multimeric grain-marked micelles with Fe_3_O_4_ inner core and outer multi grain micelle PLLA-b-PEG-Ma	98	KB tumor-bearing nude mice *in vivo*; KB cells line *In vitro*	[94]
**Passive** targeting	PpIX	PEG-PLA	30	H2009 lung cancer cells	[85]
Polymer degrades at site of action due to presence of lipase	*m*-THPC	mPEG750-b-oligo(ɛ-caprolactone)5 (mPEG750-b-OCL5) with a hydroxyl, benzoyl or naphthoyl end group		*In vivo*; *In vitro* on head & neck squamous carcinoma cell line UM-SCC-14C	[83]
Study the effect of the length of hydrophobic units, PPO, on phototoxicity and solubility	ZnPc	Poloxamine polymers: T304 (15-PEO unit, 17.1-PPOunit) T904 (60.9-PEO unit, 69.3-PPO unit) T1107 (238.6-PEO unit, 77.6-PPO unit) T1307 (286.4-PEO unit, 93.1-PPO unit)	2.7 4.9 13.9 47.6	KB cells; Increase in phototoxicity and ZnPc solubility by increasing PPO unit	[65]
**Passive** targeting	Xanthene dye erythrosine B (ERY)	CTAB (cationic) micelle SDS (anionic) micelle		logP of 0.46 (hydrophilic dye)	[95]
Tri-block copolymer	Gn-DPcZn	Polyion micelle; Amphiphilic triblock copolymer; PLL-b-PEG-b-PLL		High stability *in vivo*	[96]
**Passive** targeting	Pheophorbide	PEO(2000)-b-PCL(2800) PEO(5000)-b-PCL(4000) PEO(2400)-b-PDLLA(2000) PEO(3100)-b-PS(2300)	20–30	HCT-116 human colorectal carcinoma	[97]

## 3. Increased ^1^O_2_ Production

### 3.1. Incorporation of Heavy Atoms

**Figure 15:**
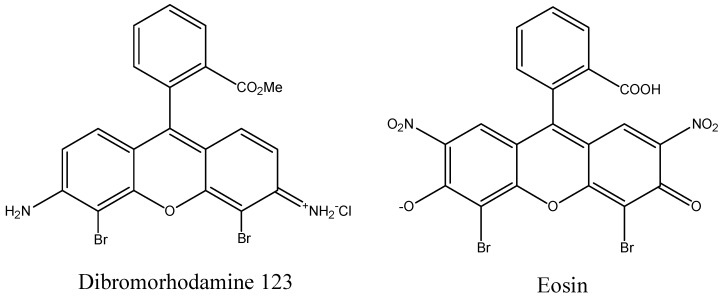
Eosin and dibromorhodamine structures containing heavy atoms.

**Figure 16:**
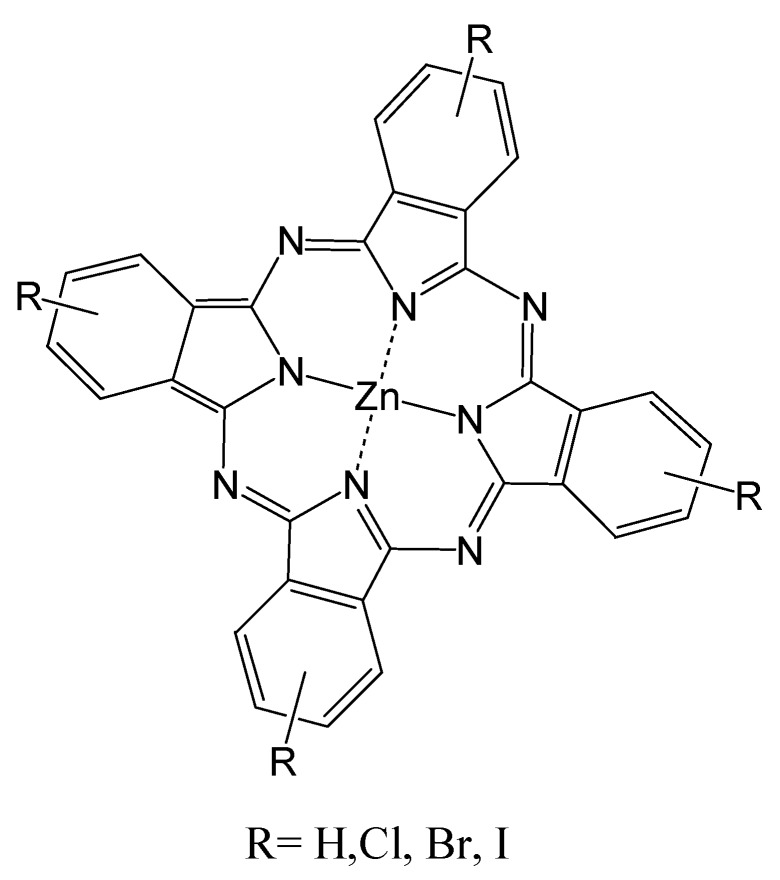
Halogenated Zn-Pc structures for enhanced ^1^O_2_ formation efficiency.

The ability to produce satisfactory levels of ^1^O_2_ is a critical characteristic of PDT sensitizers and correlates with increasing the triplet excited state lifetime of a PS [98,99]. Increased triplet state lifetime can occur by increasing ISC efficiency. One way to increase ISC is by incorporating heavy atoms into the sensitizer structure, as illustrated for eosin and rhodamine in Figure 15 [100,101,102]. A closed-shelled diamagnetic metal is better suited than an open-shelled paramagnetic metal because the latter shortens the triplet lifetime and makes certain chromogens photo-inactive. Halogenation of the rings in the phthalocyanine (Pc) system, Figure 16, also increases the ^1^O_2_ quantum yield, which can be explained using the principles of spin-orbit coupling. In this regard a bathochromic shift in absorption maxima as well as an slight increase in ^1^O_2_ production was observed by substitution of H with halogens and follows the order of Cl < Br < I. Temperature, media viscosity, and oxygen concentration can also influence ^1^O_2_ production [39]. The development of complexes contain Ir is another approach to increasing ^1^O_2_ levels. For instance, cyclometalated Ir(III) complexes such as fac-Ir(ppy)_3_ 1 (ppy = 2-phenylpyridine) and fac-Ir(tpy)_3_ 2 (tpy = 2-(4′- tolylpyridine)) have unique photophysical properties as phosphorescence materials, since they possess high luminescent quantum yields and have relatively long phosphorescent lifetimes (τ ~ μs) [49]. The highly efficient spin–orbit coupling of Ir(III) metal ion in the complexes promotes ISC to the triplet state. Consequently, these complexes exhibit strong phosphorescence even at room temperature. The authors also used DFT calculations to show that protonation of the pyridyl groups narrows the HOMO–LUMO energy gap and induces a red-shift in the emission wavelength [49].

**Figure 17:**
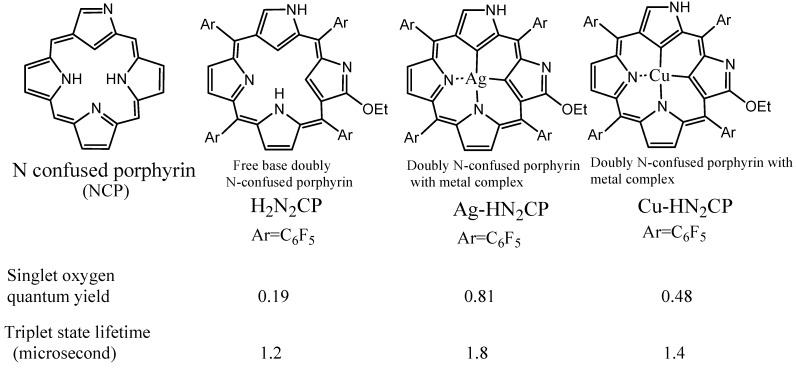
Properties of N-confused porphyrin (H_2_N_2_CP) with, and without, metal atoms.

**Figure 18:**
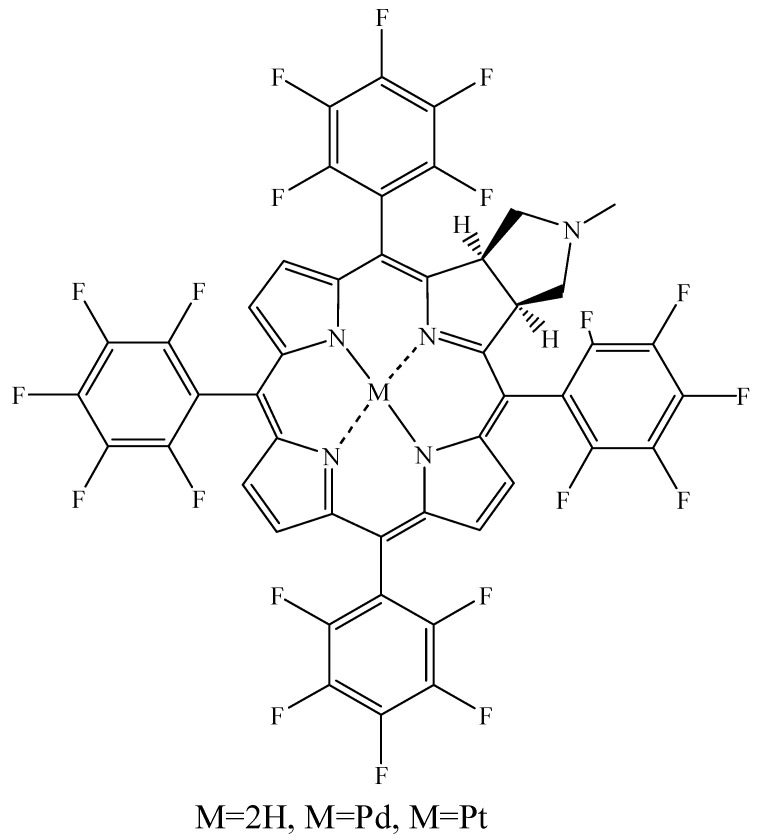
Pd- and Pt-complexed 5,10,15,20-tetrakis (pentafluorophenyl) porphyrin structures.

Incorporation of Ag (III) into *N*-confused porphyrin (H_2_N_2_CP) can also increase the efficiency of ^1^O_2_ generation, Figure 17, by increasing ISC efficiency to the triplet state [103]. Incorporating heavy metals in 5,10,15,20-tetrakis(pentafluorophenyl) porphyrin, Figure 18, increased ^1^O_2_ quantum yield from 0.28 to 0.89 and 0.92 for Pd(II) and Pt(III), respectively [104].

**Figure 19:**
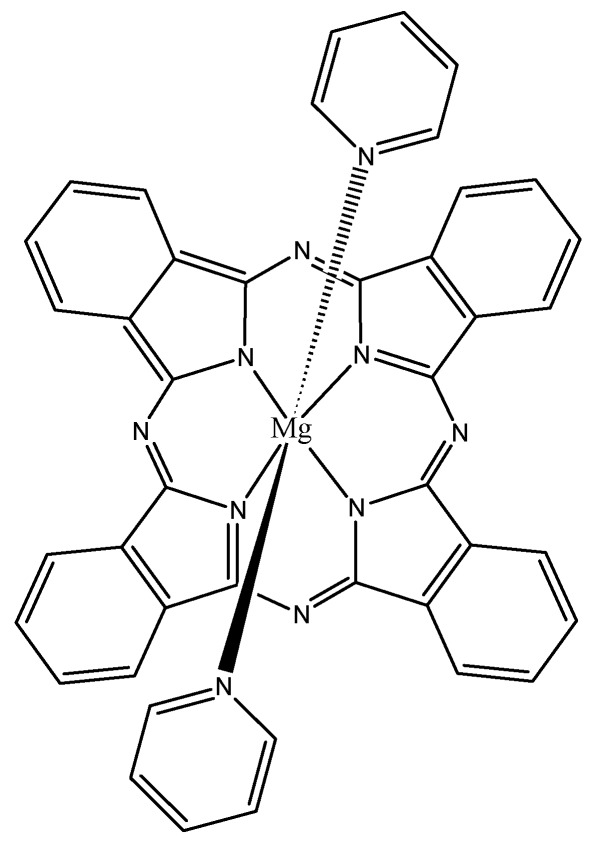
A Mg tetrabenzoporphyrin having high ^1^O_2_ quantum efficiency.

Up to a 70% increase in ^1^O_2_ quantum efficiency was reported for Mg tetrabenzoporphyrin containing a pyridine ligand, Figure 19 [105]. Placing one or five Zn atoms into a picolylamine-based porphyrin raised the ^1^O_2_ quantum yield from the 0.5 to 0.82 and 0.92, respectively [106].

### 3.2. Isosteric Replacements

**Figure 20:**
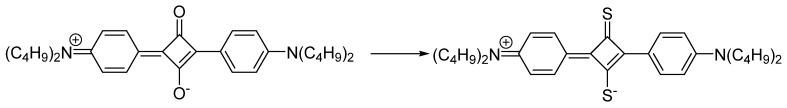
Modification of the squaraine system to increase ^1^O_2_ quantum efficiency.

Structures for enhancing intersystem crossing in polymethine-like molecules were developed, by replacement of oxygen atoms by sulfur in the squaraine system, as illustrated in the Figure 20. This replacement facilitates S_1_(nπ*)→T_1_(ππ*) and the magnitude of the resultant ISC rate constant increased ^1^O_2_ quantum efficiency [107,108].

**Figure 21:**
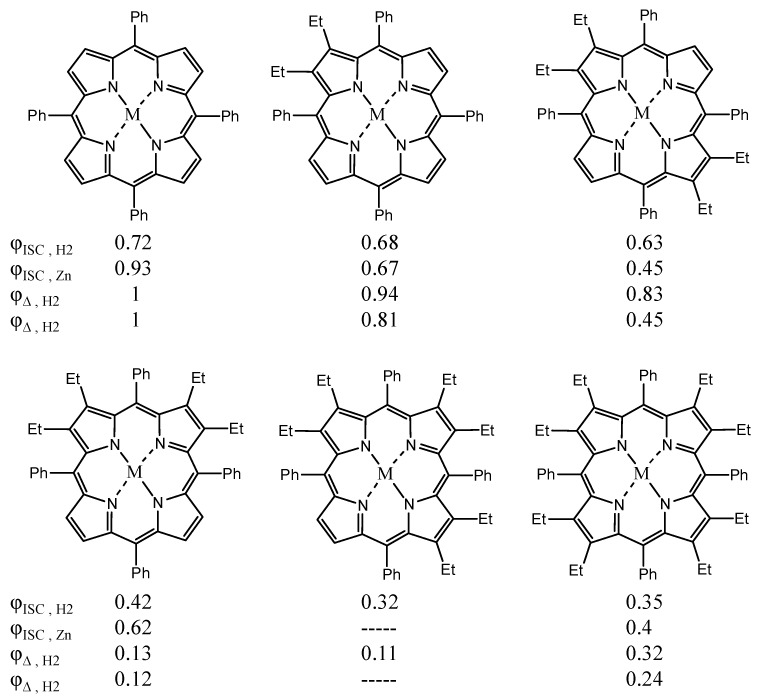
^1^O_2_ quantum efficiency values for various tetraphenylporphyrins.

In the case of porphyrin and phthalocyanine systems, the conformation and the symmetry of these molecules also affects ^1^O_2_ production [109]. In a study by Roder and coworkers, it was shown that the addition and the number of ethyl groups in tetraphenylporphyrins effected a gradual change in properties such as ^1^O_2_ production and ISC. The ISC and ^1^O_2_ quantum yield values for tetraphenylporphyrins with and without Zn are summarized in Figure 21. These measurements were conducted in DMF [110].

**Figure 22:**
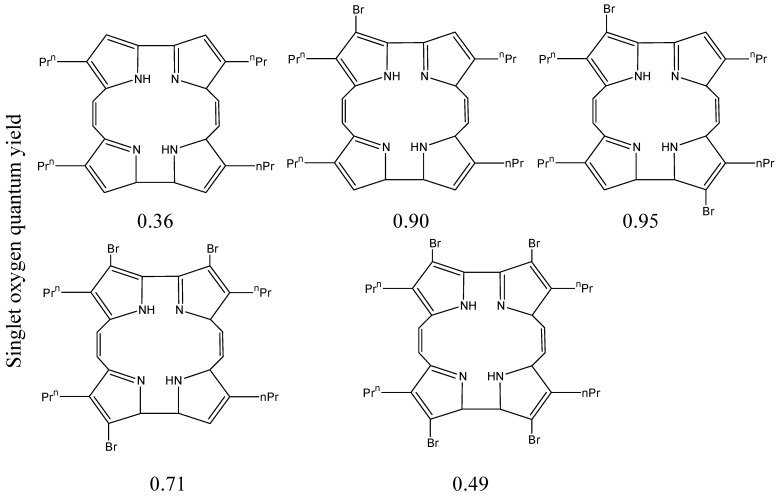
Brominated porphycene macrocycles and their ^1^O_2_ quantum efficiency.

**Figure 23:**
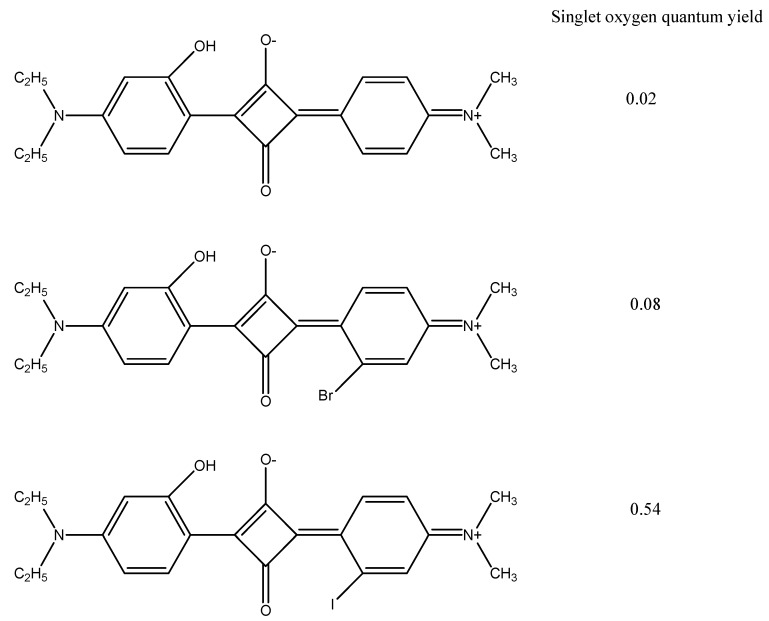
Iodinated squaraine giving enhanced ^1^O_2_ quantum efficiency.

Incorporation of 1–4 bromine atoms into the porphycene macrocycle, Figure 22, changed ^1^O_2_ efficiency from 0.36 to 0.9, 0.95, 0.71, and 0.49. It was also found that the symmetry and geometry of the molecules affected ^1^O_2_ production [111]. Asymmetrical halogenated aniline-based squaraines, Figure 23, include an iodinated derivative giving increased ^1^O_2_ production [112]. Molecules with higher symmetry have a similar singlet and ground state geometry. This similarity in geometry led to increased fluorescent quantum yield and reduced ISC efficiency.

It is believed that the use of heavy atoms to encapsulate a PS enhances ISC and thus ^1^O_2_ quantum efficiency. With this in mind, Pt(IV)- and Au-modified silica nanoparticles were developed as a delivery system for enhancing the PDT effectiveness of hypocrelin A (HA). Comparing ^1^O_2_ efficiency results from encapsulating HA with, and without, Pt- and Au- modification, indicated that including one of these heavy atoms as a dopant increases ^1^O_2_ generation [113].

**Figure 24:**
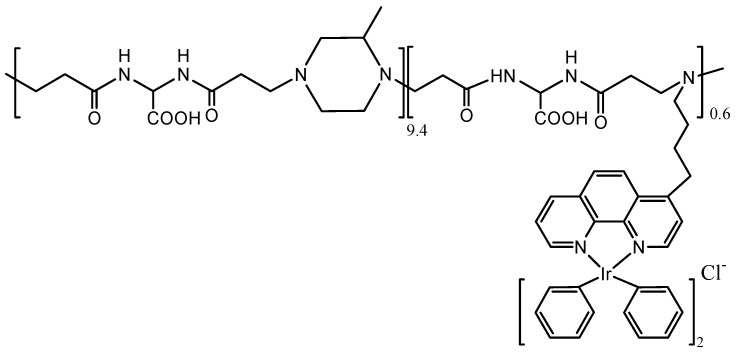
Poly(amidoamine) Ir(III) complex as a new ^1^O_2_ sensitizer.

Cyclometalated Ir complexes have grown in interest as PSs, due to recognition of their high quantum yields of triplet formation, long lifetimes of the excited triplet state (typically in the μs range), and triplet energy high enough to allow for the energy transfer process. Further, it has been shown that they are usually resistant to attack by ^3^O_2_. However, disadvantages, such as low H_2_O solubility, hinder biomedical applications of these complexes. To address this concern, the synthesis and photochemical characterization of a water-soluble Ir−PhenISA conjugate was undertaken, the results of which showed that binding to a suitable polymer improved the photophysical properties of the Ir emitters. In this regard, bis-(cyclometalated) Ir complexes appended to the polymer afforded triplet metal-to-ligand charge transfer excited states that can either radiatively decay or react with ^3^O_2_ and can then be exploited for either imaging or PDT applications. It has been found that photoluminescence can be triggered by two-photon excitation, which offers advantages over traditional one-photon excitation in terms of less cellular damage and deeper light penetration *in vivo*. Specifically, binding an Ir(III) complex (triplet emitter) to a poly(amidoamine) gave the Figure 24 conjugate [11], doubling the luminescent quantum yield and demonstrating their potential use as both cell imaging agents and PSs for ^1^O_2_ generation.

**Figure 25:**
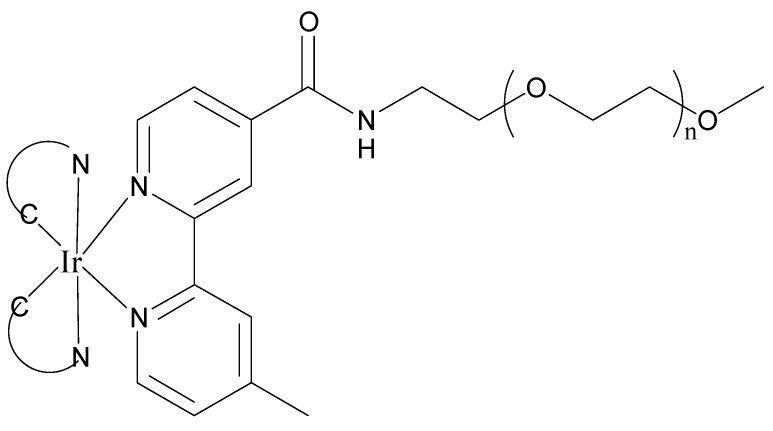
Ir(III) PEG complexes as new ^1^O_2_ sensitizer.

A group of water-soluble IR-bipyridyl complexes (Figure 25) displayed significantly reduced cytotoxicity, with half maximal inhibitory concentration (IC_50_) values being up to 10^2^ μM after a 48-h incubation, which were ~90 times larger than values from their PEG-free counterparts. It is believed that the reduced cytotoxicity originates from the PEG pendants, which prevent the complexes from interacting with intracellular DNA, proteins, and organelles [11,74,114]. The authors studied the photophysical and photochemical properties of their Ir(III) PEG complexes and their PEG-free counterparts and found that ^1^O_2_ production efficiency was closely related to the emission lifetimes of the complexes, which can be systematically controlled by changing the cyclometalating ligands. The cellular uptake efficiency of the PEG complexes was lower than their PEG-free counterparts. Since all these PEG complexes were non-cytotoxic in the dark, but exhibited considerable light-induced cytotoxic activity, they have potential to serve as efficient PS for PDT. It was also confirmed that the PEG complexes were localized in the mitochondrial region, facilitating efficient oxidative damage of this cellular organelle and causing necrotic cell death upon light excitation [74].

### 3.3. Two Photon Systems

**Figure 26:**
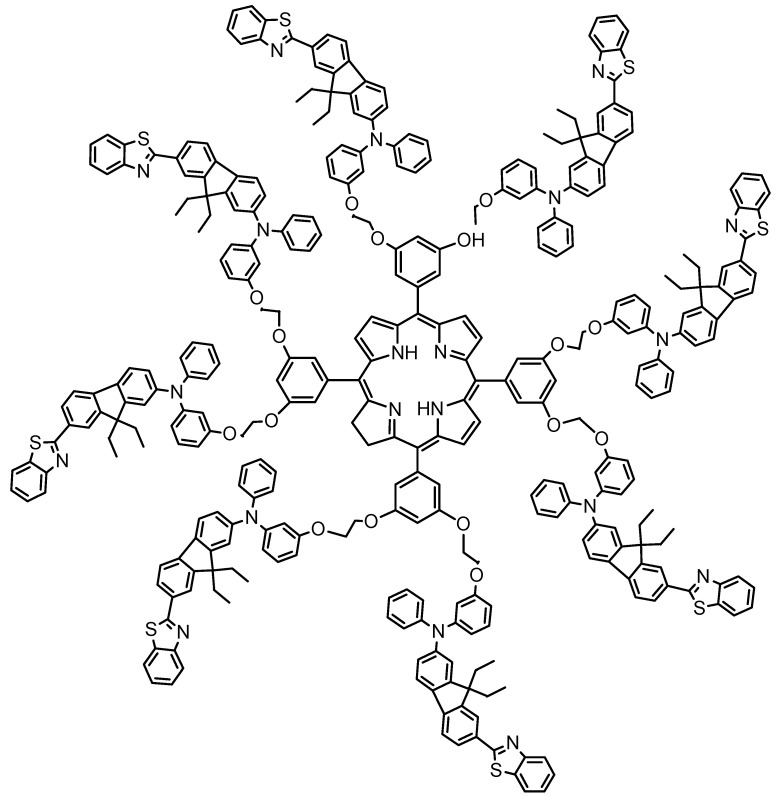
A light harvesting dendrimer having fluorescence resonance energy transfer.

Approaches to increasing triplet state efficiency include designing molecules capable of absorbing two photons simultaneously in the same quantum event. The associated electron donor (D) acceptor (A) combinations are linked through a conjugated linker (π-bridge) and are symmetrical or asymmetrical, forming (D-π-D) or (A-π-A), (D-π-A)[115], (A-π-D-π-A) and (D-π-A-π-D) structures [116]. Two photon absorption (TPA) is very attractive for biological applications which require NIR absorbing systems [117]. One approach to increasing TPA efficiency is to incorporate a light-harvesting dendrimer (antenna) containing chromophores that funnel the excited-state energy of the PS without changing the desired photochemical and photophysical properties of the porphyrin, Figure 26 [117].

Two-photo activated PDT (2-γ PDT) provides the potential for treating deeper tumors and/or enhancing tumor targeting. Pyropheophorbide-a methyl ester (MPPa) has been used to study one- and two-photon activated PDT and it was found that femtosecond laser pulses at 674nm provides 1-γ PDT efficacy against cervical, lung, and ovarian cancer cells. It was also shown that MPPa can be activated by a 120 fs laser at 800 nm at a light dose causing no detectable phototoxicity [118].

**Figure 27:**
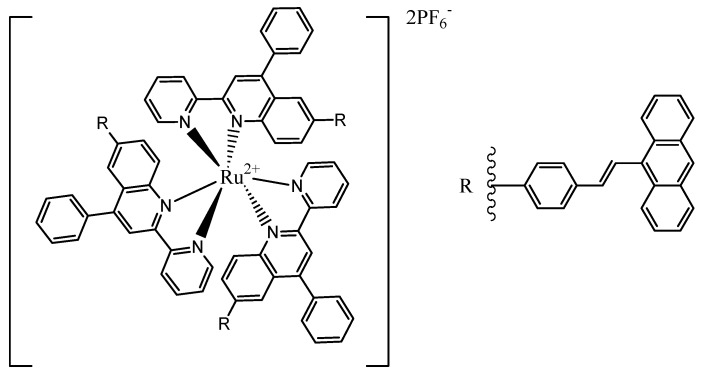
Structure of [Ru(LP1)_3_][PF_6_]_2_.

The addition of π-conjugation to Ru(II)-based PSs has been studied. The sensitizing ability of the complex increased by addition of one or two pyridine-quinoline hybrid systems to an anthracene unit. While the addition of two heteroaromatic ligands had a beneficial effect, adding a third group to the complex reduced photosensitization which is due to the bulkiness of the whole system, Figure 27 [119].

### 3.4. Triplet PSs

Another approach to increasing ^1^O_2_ production involves the use of triplet PSs. Triplet PSs have applications in catalysis of organic reactions, light-induced hydrogen production, and PDT [120]. To produce a triplet PS, population of the first triplet excited state is required which can be done by ISC.

**Figure 28:**
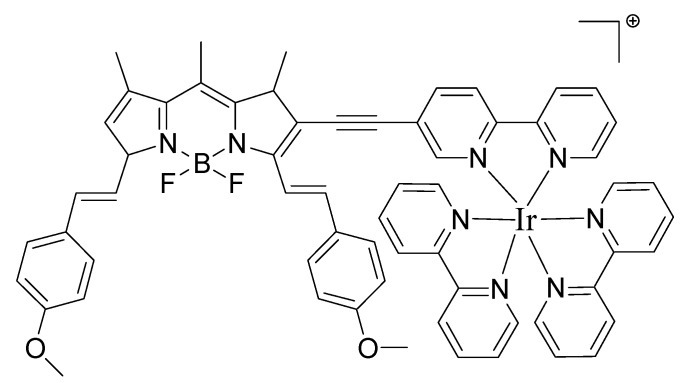
Structure of cyclometalated Ir(III) complex.

Cyclometalated Ir(III) complexes with styrylBODIPY ligands and showing NIR absorptions/emissions have been reported. The complexes were used as triplet PSs for ^1^O_2_ mediated photooxidations. In general, cyclometalated Ir(III) complexes having short absorption wavelength, weak absorption of visible light, and short-lived triplet excited states, are not suitable for PDT application PDT because strong absorption of visible light and long-lived triplet excited states are preferred [120,121]. Consequently, a key goal has been to develop new complexes that show strong fluorescence as well as satisfactory ISC. To develop transition metal complexes showing strong visible light absorption and long-lived triplet excited states bulky organic fluorophores have been attached to the coordination center using a C–C triple bond as the *p*-conjugation linker. In this way, the heavy atom effect of Ir(III) can be maximized and the excitation energy can be efficiently channeled to the triplet excited states thus enhancing the PDT applicability of Ir(III) complexes. More importantly, the absorption as well as the emission wavelengths can be extended to the NIR spectral region (644–729 nm). This approach led to heteroleptic cyclometalated Ir(III) complexes containing BODIPY (Figure 28) exhibiting strong NIR absorption, strong NIR fluorescence (700–800 nm), and long-lived triplet excited states (92–156 ms). The photophysical properties of the complexes and the NIR light-harvesting ligands were studied using steady state and time-resolved absorption/emission spectroscopy, as well as DFT calculations [122].

**Figure 29:**
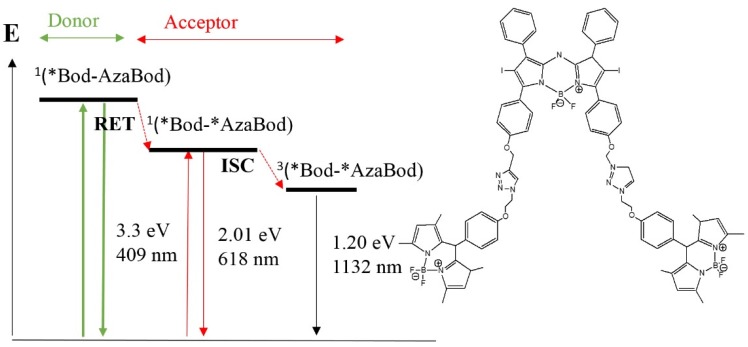
Example of resonance energy transfer (RET) and ISC for a BODIPY/aza-BODIPY based PS.

It has been reported that photooxidation enhancement can be accomplished through intramolecular resonance energy transfer (RET). In a study by Zhao and coworkers, Bodipy triad triplet PSs (Figure 29) were developed by connecting an unsubstituted styryl-BODIPY (energy donor), with λ_max_ of 409, to bisiodo aza-BODIPY (intramolecular energy acceptor) through click chemistry. The presence of additional absorption bands in the visible regions enhanced light absorption in the visible region. Therefore the energy of the donor can be transferred to the acceptor via ISC. Aza-BODIPY with a much larger absorption band functions as a spin convertor to produce the triplet excited state [123].

**Figure 30:**
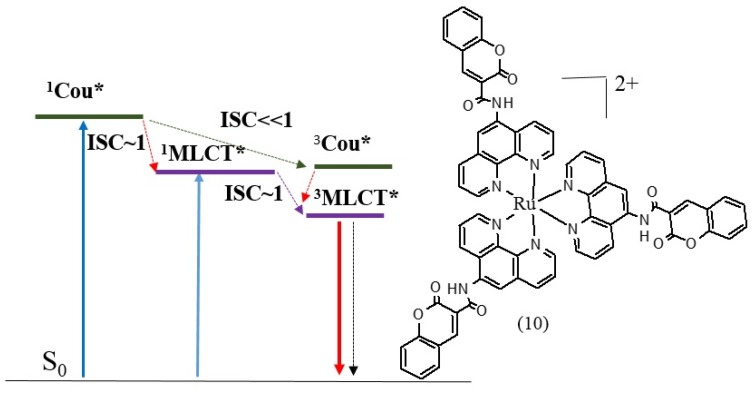
Energy level diagram showing Ru(II) complex having a light-harvesting coumarin ligand.

In a closely related report, Zhao and coworkers summarized the literature pertaining to design approaches for triplet PSs [120]. An example was the design of complexes having large molecular absorptivity values (ε_max_) in the visible spectrum and long-lived triplet excited states. Energy funneling based triplet PSs that show broadband absorptions in the visible spectrum were discussed, as a way to enhance triplet PS performance. A key goal was to develop new triplet PSs by establishing correlations between ISC and molecular structures. This report included Ru(II) polyimine triplet PSs structures such as the coumarin-based Ru(II) complex in Figure 30, which gave an absorption at 341 nm having ε_max_ ~ 79,000 *versus* an ε_max_ = 14,000 when the coumarin moiety was replaced by *N*-acetyl group. It was shown that the energy level of the ligand localized excited state (^1^IL) was higher than the ^1^MLCT (metal to ligand charge transfer) state, providing efficient energy transfer or internal conversion from the coumarin moiety to the coordination center. In addition, the energy level of the ^3^IL of coumarin is significantly higher than the ^3^MLCT; consequently the phosphorescence lifetime and the quantum yield were not adversely affected by the ligand.

**Figure 31:**
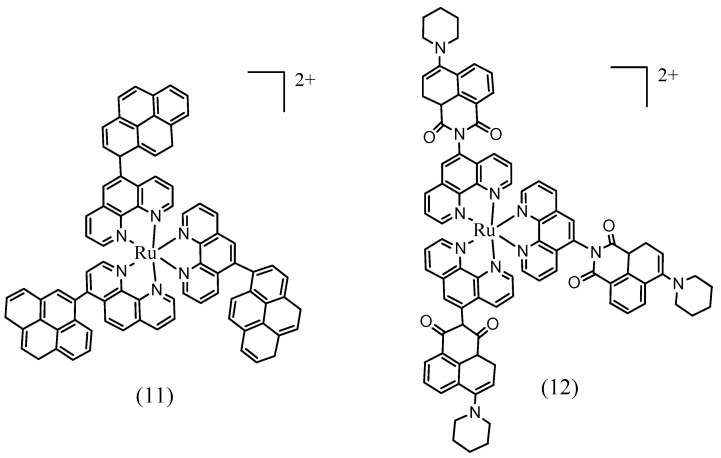
Example Ru(II) based phen complexes having close ^3^MLCT and ^3^IL states.

Ru(II) complexes having pyrene and naphthalimide appendages were also reported [120,124,125]. Attachment of pyrene groups to the phenanthroline (Phen) ligand led to enhancement of the absorption in the 300–350 nm region. In this case, the ^3^IL energy level is close to the ^3^MLCT and the triplet state lifetime is enhanced. Similarly, a long lived triplet state was observed for the Ru(II) polyimine complex having a 4-piperidinyl-1,8-naphthalimide group attached to the Phen ligand (Figure 31).

Another approach to increasing the triplet lifetime involved having the ligand and coordination center connected through π conjugation such as a C≡C moiety [120,122]. The properly chosen ligand can also enhance λ_max_ but the ligand cannot be very bulky since this would decrease ISC efficiency.

**Figure 32:**
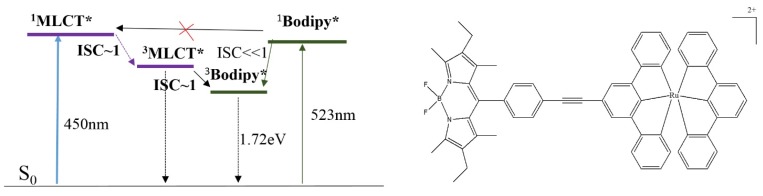
Energy diagram for a Ru(II) complex having BODIPY as a visible light harvesting ligand, showing energy transfer is unlikely.

Figure 32 shows that the ^3^MLCT state of the coordination center (1.94 eV) is higher than the ^3^IL state energy level of Bodipy (1.72 eV). Therefore the phosphorescence of the ^3^MLCT state is quenched by ^3^MLCT→^3^IL and the triplet state is mostly localized on the Bodipy unit. In this case, energy transfer from ^1^IL→^1^MLCT is not likely to happen since the ^1^IL is located below ^1^MLCT. Therefore the strong visible absorptions of the Bodipy (523 nm) and ε_max_ (66,000) do not aid triplet excited state production.

It is known that charge transfer can decrease ^1^O_2_ production by quenching of the excited state [126]. However, complexes such as Ru(II) bipyridine and related compounds are good PSs of singlet oxygen in spite of the charge transfer (CT) nature of the lowest excited state of these compounds. This is because of the relatively long lifetime of the triplet metal-to-ligand charge transfer states, ^3^MLCT, of many Ru(II) coordination compounds that make these excited states susceptible to quenching by oxygen. It is been reported that the MLCT absorption mainly produces ^1^MLCT that undergo ISC and populate ^3^MLCT [127].

## 4. Conclusions

A substantial number of porphyrinoid and non-porphyrinoid PSs both hydrophobic and hydrophilic have been published, some of which show promise as PDT agents. The key goal of much of this work was to identify approaches to PSs having high selectivity for tumors than healthy tissues and/or enhance ^1^O_2_ production. In results strictly comparing the advantages and disadvantages of hydrophobic *vs* hydrophilic PS dyes, is appears that amphiphilic PSs provide the way forward in this area. To increase the selectivity of PSs for malignant tissues, the use of PS-nanoparticle combinations has provided an interesting and potentially viable approach. In this regard, micelles/polymeric micelles having the ability to transport hydrophobic and amphiphilic PSs to mitochondria have been developed. To increase ^1^O_2_ production, systems containing heavy atoms and having enhanced ISC and two-photon absorption properties are of considerable interest. Notably, the PDT field has attracted photochemists, organic and inorganic chemists, color chemists, pharmacologists, and more recently, laser technologists, among others, to this still fascinating opportunity to have an effective, non-invasive resource for cancer treatment.
